# Cycling of the *E. coli* lagging strand polymerase is triggered exclusively by the availability of a new primer at the replication fork

**DOI:** 10.1093/nar/gkt1098

**Published:** 2013-11-13

**Authors:** Quan Yuan, Charles S. McHenry

**Affiliations:** Department of Chemistry and Biochemistry, University of Colorado, Boulder, CO 80309, USA

## Abstract

Two models have been proposed for triggering release of the lagging strand polymerase at the replication fork, enabling cycling to the primer for the next Okazaki fragment—either collision with the 5′-end of the preceding fragment (collision model) or synthesis of a new primer by primase (signaling model). Specific perturbation of lagging strand elongation on minicircles with a highly asymmetric G:C distribution with ddGTP or dGDPNP yielded results that confirmed the signaling model and ruled out the collision model. We demonstrated that the presence of a primer, not primase *per se*, provides the signal that triggers cycling. Lagging strand synthesis proceeds much faster than leading strand synthesis, explaining why gaps between Okazaki fragments are not found under physiological conditions.

## INTRODUCTION

The *Escherichia coli* chromosome is replicated by a dimeric DNA polymerase III holoenzyme (Pol III HE) in a reaction, where replication is coupled between the leading and lagging strands (1–3). Like all replicases, the Pol III HE is tripartite with a specialized replicative polymerase (Pol III, αεθ), a sliding clamp processivity factor (β_2_) and a clamp loader (DnaX complex, DnaX_3_δδ’χψ) [reviewed in (4,5)]. The leading strand is highly processive, capable of synthesizing products of at least 150 000 bases *in vitro* without dissociating (6–8). In a coupled reaction, the lagging strand polymerase must cycle rapidly during the synthesis of a series of Okazaki fragments. Thus, a signal must exist that triggers the lagging strand polymerase to switch from a highly processive state to one that can rapidly cycle to the next primer synthesized at the replication fork.

Two models have been proposed for the source of the signal that triggers cycling. In the ‘signaling’ model, it was proposed that synthesis of a new primer by primase triggers the lagging strand polymerase to cycle, even if the Okazaki fragment being synthesized is incomplete (9). Evidence for this model first arose in early replication fork reconstitution experiments that demonstrated that if Pol III was diluted below ∼1 nM, gaps were detected between Okazaki fragments (9). This suggested that the signal that triggered cycling was not dependent upon Okazaki fragment completion. DnaG primase synthesizes primers by reversible association with the DnaB helicase (10). Thus, primase concentration determines the frequency of priming and the length of Okazaki fragments. Together, these observations led to the proposal that primase set the timing of events at the replication fork and provided the signal of lagging strand polymerase release and recycling to the next primer (10).

Further support for the signaling model derived from the observation that long leading strands could be synthesized, providing long templates for lagging strand synthesis, in the absence of primase. Upon addition of primase, the first Okazaki fragment could have a length up to 100 kb in the absence of a signal causing the polymerase to cycle, yet the first Okazaki fragment was of normal length, again suggesting the signal is independent of Okazaki fragment completion (11).

An alternative model, the ‘collision’ model was first proposed for bacteriophage T4 (12), and later extended to posit that the *E. coli* lagging strand polymerase was triggered to recycle by colliding with the preceding Okazaki fragment (13) or by approaching the preceding Okazaki fragment (14). A mechanism in which τ senses the conversion of a gap to a nick and competes with the β processivity factor for binding to the C-terminus of the Pol III α subunit was proposed (15,16). However, it is now realized that the C-terminus of the Pol III α subunit does not contain the binding site required for a processive interaction with β either *in vitro* or *in vivo* (17). Instead, the β-binding site is internal (17–20). The C-terminus of Pol III α provides the τ-binding site (17,21).

An alternative proposal was made that the OB fold within α might sense the disappearance of single-stranded DNA in advance of the primer terminus on the lagging strand (14). Consistent with this hypothesis, the ssDNA binding portion of Pol III was localized to a C-terminal region of α that contains the OB fold element (22), which may be near the template in a crystal structure of a complex of Pol III α with primed template (23). However, the electron density was not sufficient to come to a firm conclusion (23). A test of the importance of the OB fold motif was made using a mutant in which three basic residues located in the β1–β2 loop were changed to serine (14). The processivity of the mutant polymerase was decreased by the β1–β2 loop mutations, an effect that was rescued by the presence of the τ complex (14). The latter observation would seem to suggest that although the OB fold contributes to ssDNA affinity and processivity, it is not the processivity sensor, or at least that the residues mutated are not the key interactors. A mutation in a true processivity switch would be expected to be defective in the intact replicase, not just the isolated core polymerase.

The evidence behind the collision model was primarily based on modulation of binding affinities. However, the relevant issue is whether the process is kinetically competent to support physiological rates of DNA replication. Determinations of the rates and extents of Pol III dissociation upon completion of an Okazaki fragment (24) agreed with models proposing that dissociation is enhanced upon adding the last nucleotide to a convert a gap to a nick (13), but not with models that posit that just reducing the size of the gap between Okazaki fragments is sufficient (14). However, the half-life of Pol III HE on nicks upon Okazaki fragment completion is ∼2 min, ∼1000-fold too slow to support physiological rates of cycling during lagging strand DNA replication (24). Thus, the collision model might be involved in slow release reactions that occur at sites other than the replication fork, such as long patch DNA repair or mismatch repair, but it is too slow to support DNA replication at the fork.

In both the bacteriophage T7 and T4 replication systems, support has been obtained for the signaling model (25,26). The collision model is thought to serve as a backup in these systems, but, to our knowledge, it has not been subjected to a direct test for kinetic competency.

In this study, we sought to apply a rigorous test of the signaling model in a coupled rolling fork replication system that was reconstituted in the presence of high template concentrations so that DnaB and Pol III concentrations could be set as substoichiometric and limiting. This eliminated the possibility that exogenous polymerase or helicase might be acting on products after their initial synthesis. Large 409 nt minicircles with an asymmetric G:C composition were used, enabling specific perturbation of the extent and rate of Okazaki fragment synthesis by the dGTP analogs, ddGTP and dGDPNP, respectively. Opposing predictions are made by the collision and signaling models when lagging strand synthesis is perturbed, permitting us to distinguish which model is used at the replication fork in *E. coli*.

## MATERIALS AND METHODS

### Proteins

DnaB_6_ (27), DnaG (28), SSB_4_ (28), β_2_ (29) and Pol III (30) were purified as described. PriA, PriB, DnaT and DnaC were expressed in *E. coli* BL21 DE3 pLysS and purified by modifications of procedures of Marians (27) that are described under Supplementary Experimental Procedures.

Pol III* was expressed in *E. coli* strain BLR [*F*^−^ *ompT hsdS_B_*(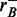 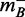) *gal dcm Δ*(*srl-recA*)*306::*Tn10 (Tet^R^)] containing the plasmid pHOC 2.6.1, and the purification is described under Supplementary Experimental Procedures. This plasmid contained the structural genes for the components of Pol III* expressed behind an IPTG-inducible P_A1_ promoter in the order: *dnaQ*, *holE*, *dnaE*, *holC*, *holD*, *holB*, *holA* and *dnaX*. The spacing between genes was kept to a minimum and, where possible, translational terminators were overlapped with translational initiators to permit translational coupling. The parent plasmid was pDRK-N(M) (30). pDRK-N(M) contains the IPTG-inducible P_A1_ promoter, a colE1 replication origin and an ampicillin resistance gene. The *dnaQ*, *holE* and *dnaE* genes came from the plasmid pHN4 (31).

### Optimized rolling circle reaction

A total of 20 nM minicircle DNA template, 0.5 µM SSB_4_, 100 nM β_2_, 12 nM DnaB_6_, 100 nM DnaG, 2.5 nM Pol III*, 160 nM PriA, 50 nM PriB_2_, 333 nM DnaT_3_ and 108 nM DnaC were incubated with 5 µM ATPγS, 200 µM CTP, 200 µM UTP and 200 µM GTP for 5 min at 30°C. The concentration of DnaG used was on the plateau of the titration, 4-fold greater than the optimum selected. It was used to adjust the length of Okazaki fragments to the physiological range of 1–2 kb.

The reaction buffer was 10 mM magnesium acetate, 70 mM KCl, 50 mM Hepes (pH 7.5), 100 mM potassium glutamate, 20% glycerol, 200 µg/ml bovine serum albumin, 0.02% Nonidet P-40 and 10 mM dithiothreitol. The reaction was started by addition of 1 mM ATP and 100 µM dNTPs. After 3 min, α-[^32^P] dCTP or dGTP was added to allow quantification of leading or lagging strand synthesis, respectively. The reaction was quenched with either 83 mM EDTA (final concentration) for scintillation counting or an equal volume of stop mix [40 mM Tris–HCl (pH 8.0), 0.2% SDS, 100 mM EDTA and 50 µg/ml proteinase K] for gel electrophoresis after 5 min. For the analysis of the size of lagging strand products, samples were subjected to alkaline agarose gel electrophoresis.

### Nucleotide analogs

dGDPNP was custom synthesized for this project by TriLink Biotechnologies.

### Quantification of DNA synthesis

DNA products were quantified by TCA precipitation and scintillation counting as described for strand displacement replication assays (32).

### Determination of Okazaki fragment length

The lengths of Okazaki fragments (*L*) were determined by a method that removed the bias of more radioactivity being incorporated into longer products using *L* = ∑(*L_i_·n_i_*)/∑*n_i_*. *n_i_* is the relative molar amount of the Okazaki fragments with a certain length *L_i_*. *n_i_* = density*_i_*/*L_i_*, where density*_i_* is the pixel density at *L_i_* in a lane determined using ImageQuant. Thus, *L* = ∑density*_i_*/∑(density*_i_*/length*_i_*). To exclude leading strand products, only density below 8 kb was used for calculation of average Okazaki fragment length unless otherwise indicated.

## RESULTS

### Establishing an efficient *E. coli* rolling circle replication system on tailed minicircle templates with an asymmetric G:C distribution

We chose to establish a complete PriA-dependent replication system that used defined minicircle templates to exploit multiple significant advantages such systems afford (33–35). (i) Employing defined templates with an asymmetric nucleotide composition between the two strands enables quantitation of the levels of synthesis of each strand individually by measuring nucleotide incorporation. (ii) Extreme asymmetry in strand nucleotide composition permits selective modulation of the rates of lagging strand synthesis, a feature first exploited by Benkovic *et al.* to test cycling mechanisms in the bacteriophage T4 replication system (25). (iii) Use of small DNA substrates allows higher template concentrations to be used, allowing substoichiometric helicase and polymerase concentrations. This helps avoid side reactions resulting from excess helicase acting on reaction products and prevents excess polymerase extending unused primers or filling gaps at sites remote from the replication fork. (iv) Use of the full PriA/PriB/DnaT/DnaC-dependent helicase loading system avoids the necessity of using vast excesses of helicase and permits blocking of a helicase-independent background reaction catalyzed by the strand displacement activity of Pol III HE in the presence of SSB (32,36).

We established the system using a tailed 409 bp circle initially developed for *Bacillus subtilis* rolling circle replication (37). This DNA substrate contained a 50:1 ratio of C:G in the template for lagging strand synthesis. This template is longer than those commonly employed in minicircle systems and was chosen to minimize potential steric issues at the replication fork. Modifying our previous procedure for creating this template to include a PCR amplification step permitted very large quantities to be made (Supplementary Figure S8).

**Figure 1.**
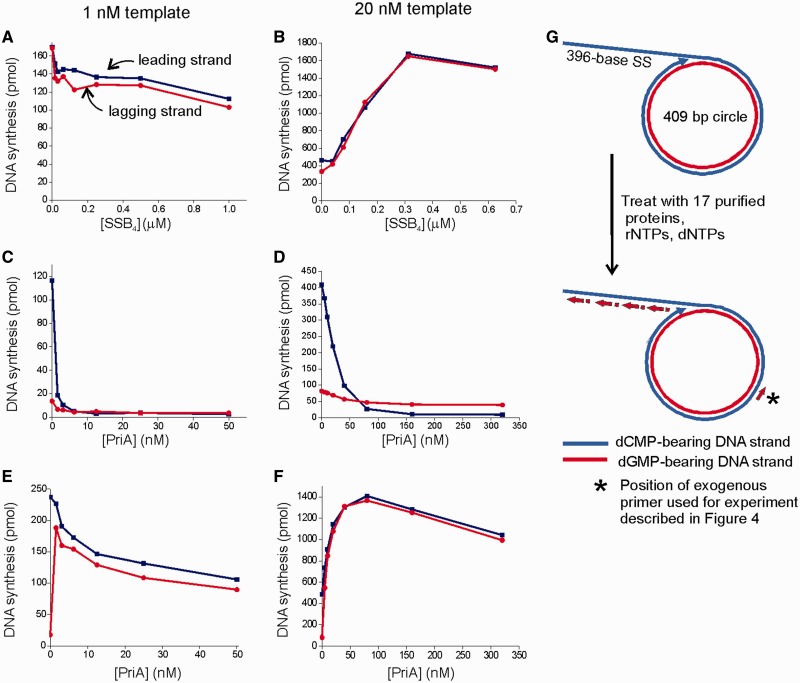
Reactions conducted at high template concentration and substoichiometric helicase and Pol III* become dependent upon PriA and SSB. (**A** and **B**) SSB titration in the presence of 1 and 20 nM DNA under optimal conditions. (**C** and **D**) PriA titration in the presence of 1 and 20 nM DNA under optimal conditions but without DnaB. (**E** and **F**) PriA titration in the presence of 1 and 20 nM DNA under optimal conditions. (**G**) Schematic of synthetic rolling circle template used for work presented in this article.

Each individual component of the reconstituted rolling circle reaction was titrated to its optimum, except DnaB_6_ helicase and Pol III* (a complex of Pol III and τ-containing DnaX complex) which were deliberately maintained at limiting, substoichiometric concentrations (Supplementary Figures S1–S3). Titrations performed at standard low (1 nM) versus high (20 nM) template concentrations revealed significant differences. We were unable to obtain efficient replication with substoichiometric helicase and polymerase in the presence of 1 nM template, presumably because we were working below *K*_D_s required for reaction components to efficiently interact (Supplementary Figure S1). An acceptably efficient reaction could be reconstituted with 12 nM DnaB_6_ and 2.5 nM Pol III* on 20 nM template (Supplementary Figure S3). We observed significant differences in the requirement for SSB between the low and high template concentration systems. In the presence of 20 nM template, SSB stimulated the reaction 4-fold. No SSB dependence was observed in the presence of 1 nM template (Figure 1A and B).

PriA serves as both the initiation protein that recognizes stalled forks and as a checkpoint protein that blocks strand displacement by the replicase in the absence of a properly assembled helicase. The effect of PriA on reactions performed at low and high template concentrations also differed in potentially important ways. As the first step of our optimization, we titrated PriA into an otherwise complete reaction that lacked DnaB_6_ to determine the level of PriA required to completely block a background reaction catalyzed by the strand displacement activity of the Pol III HE (Figure 1C and D). Reactions containing higher template concentrations required higher concentrations of PriA for inhibition of intrinsic strand displacement activity of Pol III HE. PriA was then titrated into a complete reaction that contained DnaB_6_. In reactions conducted at low template concentrations, we observed a high background level of leading strand synthesis in the absence of PriA (Figure 1E). Addition of PriA restored stoichiometric leading and lagging strand synthesis. At high template concentration, which permitted use of substoichiometric DnaB_6_ and Pol III*, equivalent levels of leading and lagging strand synthesis were observed at all levels of PriA (Figure 1F). Instead of the inhibition that we observed on 1 nM template, we observed a stimulation with an optimum near the point where helicase-independent strand displacement is abolished. These characteristics led us to have a higher level of confidence in reactions conducted using 20 nM template and substoichiometric DnaB_6_ and Pol III*, and these conditions were used for further study.

In the presence of low helicase and Pol III* concentrations, helicase loading and initiation complex formation are rate-limiting. We discovered these barriers could be overcome by pre-incubation of all components with ATPγS and CTP, UTP and GTP (Supplementary Figure S4). ATPγS supports both loading of the helicase if the other primosomal proteins are present (Manhart, C. and McHenry, C., in preparation) and initiation complex formation by the leading strand half of the dimeric replicase (38). The absence of ATP prevents translocation of loaded helicase.

### Specific blockage of Okazaki fragment synthesis before completion does not terminate overall lagging strand synthesis

**Figure 2.**
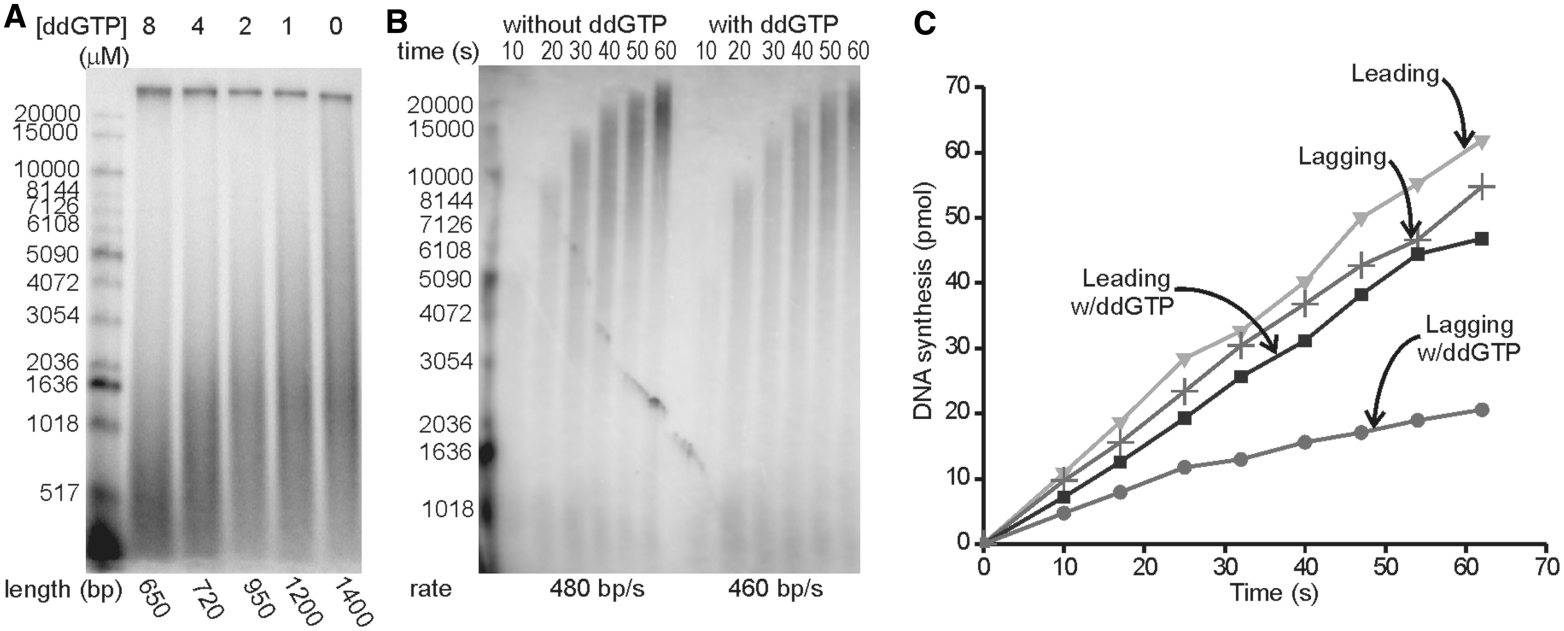
ddGTP halts Okazaki fragment synthesis before completion, but lagging strand synthesis continues. **(A)** Reactions were carried out under conditions of the optimized rolling circle reaction with ddGTP added at the same time as radiolabeled nucleotide. Okazaki fragments incorporating α-[^32^P] dGTP (10 000 cpm/pmol) were monitored by alkaline agarose gel electrophoresis. Average lengths were determined with a cutoff at 20 kb to exclude leading strand products from the quantification. **(B)** In the presence or absence of 4 µM ddGTP, an optimized rolling circle reaction was conducted as in (A) except that α-[^32^P] dCTP (20 000 cpm/pmol) and ddGTP were added at the same time as dNTPs and ATP. The 3 min elongation step before the addition of radioactive nucleotide was skipped so that the products would be short enough for accurate length quantification. Radiolabeled leading strand products were monitored on an alkaline agarose gel. The products of 10, 20 and 30 s reactions were used to calculate the rate of leading strand synthesis. **(C)** The amount of leading and lagging strand synthesis in the absence or presence of 1 µM ddGTP.

The asymmetry in G:C composition between the leading and lagging strand templates permits specifically perturbing lagging strand synthesis by the addition of low concentrations of ddGTP in the presence of the normal four dNTPs. This chain terminator would be expected to terminate synthesis before Okazaki fragment synthesis is complete on the high C content (45%) lagging strand template. The signaling model would predict efficient cycling of the lagging strand polymerase upon new primer synthesis at the fork. The collision model would predict an abrupt cessation of lagging strand synthesis, because the polymerase would never collide with the 5′-end of the preceding Okazaki fragment and be induced to cycle. Addition of increasing concentrations of ddGTP decreases the length of the Okazaki fragments observed (Figure 2A). In a control reaction, the rate of leading strand synthesis was shown to be unperturbed, consistent with the low C content of the leading strand template and the presence of the 3′→5′ proficient proofreading exonuclease that would be expected to remove low frequency ddGMP incorporation (Figure 2B).

We next measured the rate of nucleotide incorporation into the leading and lagging strands in the presence and absence of ddGTP. We observed a nearly undiminished rate of leading strand synthesis and a drop in lagging strand nucleotide incorporation to about one-half (Figure 2C). In the presence of ddGTP, the length of Okazaki fragments is reduced (Figure 2A), explaining much of the reduction of synthesis. If the collision model was the dominant model on the time scale of the experiment, one Okazaki fragment would be synthesized in <2 s and synthesis would stop, leading to complete abolition of Okazaki fragment synthesis. When these reactions were conducted in the presence of increasing ddGTP concentrations, the level of Okazaki fragment synthesis decreased, but synthesis continued at a remarkably linear rate (Supplementary Figure S5). Much of the decrease is due to shortening of the Okazaki fragments at increasing ddGTP concentrations. Thus, the molar level of Okazaki fragment synthesis does not decrease to the same extent as nucleotide incorporation.

### Selectively slowing the rate of lagging strand synthesis with dGDPNP results in cycling to the next primer before Okazaki fragment completion, consistent with the signaling model

The signaling model predicts that the Okazaki fragments synthesized in the presence of ddGTP would have gaps between them that corresponded proportionally to the amount that they were shortened. We were unable to find a suitable polymerase that could completely exonucleolytically remove incorporated ddGMP without strand displacement to test this hypothesis. Hence, we switched to the use of another perturbant of lagging strand synthesis, dGDPNP, which offered its own unique advantages. dGDPNP exhibits a much higher *K*_m_ than dGTP (40 µM versus 2 µM, respectively) (Supplementary Figure S6A and B). This allowed us to adjust the rate of lagging strand synthesis by decreasing the concentration of dGDPNP without going below the concentration of the normal dNTPs in the reaction and creating artifacts from nucleotide depletion.

Measurement of the rates of primer extension on M13 templates was used as a surrogate for a template that required the incorporation of dGMP for elongation, like the lagging strand template in our model minicircle system. Substitution of 240 µM dGDPNP for saturating dGTP led to a 10-fold decrease in elongation rate, suggesting a slowing of the chemistry step of the polymerase reaction in addition to the *K*_m_ effect (Supplementary Figure S6C). Reduction of dGDPNP to 30 µM decreased the elongation rate ∼25-fold relative to dGTP. Control reactions for the rate of leading strand synthesis indicated the lack of a significant perturbation, consistent with the C-deficient leading strand template (Supplementary Figure S6D).

**Figure 3.**
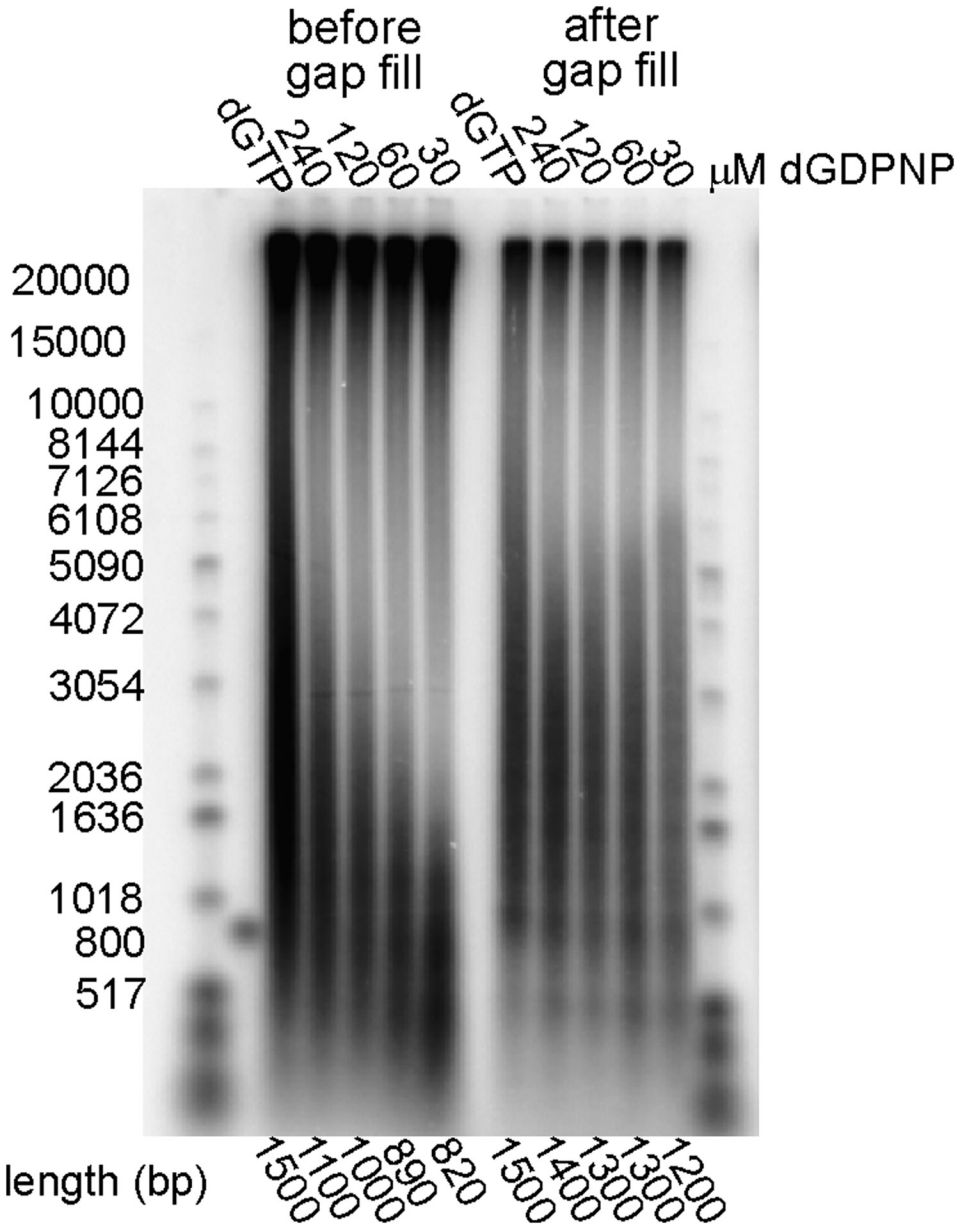
Replacement of dGTP with dGDPNP leads to short Okazaki fragments followed by large gaps. An optimized rolling circle reaction with 20 mM template was carried out in the presence of 100 µM dGTP or the indicated concentrations of dGDPNP. Products were extracted with phenol–chloroform, precipitated by isopropanol and incubated with 100 µM dNTPs, 0.2 U Phusion polymerase and α-[^32^P] dATP (12 000 cpm/pmol) at 72°C for 15 min. The products before and after the gap fill assay were monitored by alkaline agarose gel electrophoresis.

Reduction in the concentration of dGDPNP in the presence of the other three normal dNTPs resulted in a marked decrease in the length of Okazaki fragments produced (Figure 3). Deproteinization and treatment of the rolling circle reaction products with Pfusion DNA polymerase results in extension of all the shortened Okazaki fragments to approximately the same length (Figure 3). In control experiments, we demonstrated that this polymerase does not significantly strand displace (Supplementary Figure S9). The results suggest that Okazaki fragments synthesized during balanced synthesis do not have gaps between them while those synthesized in the presence of 30 µM dGDPNP have ∼400 nt gaps. Length quantification in these experiments used a normalization procedure that removed the bias of longer products containing more radioactive nucleotide. Thus, the numbers presented represent the molar mean length.

### The presence of dGDPNP does not significantly influence the frequency of primer synthesis or primer utilization

It is possible that the length of Okazaki fragments could be reduced by more frequent primer synthesis or an increased rate of primer utilization. We investigated this possibility using gel assays to quantify the percentage of unelongated primers to determine primer utilization (39) (Supplementary Figure S7). Incorporation assays, quantifying the ratios of radiolabeled GMP and UMP incorporated relative to dCMP, were used as a measure of priming frequency. We observed a modest decrease in primer utilization (from 66% to 50%) upon substitution of dGDPNP for dGTP (Table 1). The number of GMP and UMP residues/Okazaki fragment increased from 8 to 10 (Table 1). Thus, the decrease in Okazaki fragment size in the presence of dGDPNP cannot be attributed to perturbations in priming.

**Table 1.** Determination of primer utilization and priming frequency

	G + U in elongated primers^a^	G + U in unelongated primers^a^	dC in leading strand product^a^	Primer utilization efficiency^b^	Average primer utilization efficiency	G + U in elongated primer/dNMP in leading strand^c^	Average of preceding column	Gap-filled Okazaki fragment (nt)^d^	Number of G + U/Okazaki fragment (nt)^e^
100 µM dGTP	650	380	54 000	63%	66 ± 3%	0.0054	0.0051 ± 0.0005	1500	8 ± 1
	630	290	53 000	68%		0.0053			
	810	380	82 000	68%		0.0044			
240 µM dGDPNP	460	470	44 000	49%	60 ± 11%	0.0047	0.0059 ± 0.0020	1400	8 ± 3
	820	330	45 000	71%		0.0081			
	600	420	57 000	59%		0.0048			
30 µM dGDPNP	360	410	23 000	47%	50 ± 4%	0.0070	0.0081 ± 0.0016	1200	10 ± 2
	380	400	23 000	49%		0.0074			
	540	420	25 000	56%		0.0098			

^a^Relative content determined as described under Supplementary Experimental Procedures.

^b^Utilization efficiency = G + U in elongated primers/(G + U in elongated + unelongated primers).

^c^G + U in elongated primer/dNMP in leading strand = G + U in elongated primers/(dC in leading strand/fraction dC in leading strand).

^d^Values taken from Figure 3 after gap fill.

^e^Number of G + U/Okazaki Fragment = G + U in elongated primer/dNMP in leading strand × gap-filled Okazaki fragment length (nt).

### The signal for cycling of the lagging strand polymerase is the availability of a new primer at the replication fork and is not dependent upon the presence of DnaG primase

Coupled with the previous observation that the rate of release of the Pol III HE upon collision with the 5′-end of the preceding Okazaki fragment does not provide a kinetically competent pathway (24), the preceding experiments suggest that cycling of the lagging strand polymerase is regulated by synthesis of a new primer at the replication fork as initially proposed by Marians (9). With the new tools developed for this work, we sought to determine whether this signal was provided by the action of DnaG or whether just the availability of an annealed primer at the fork was sufficient. Previous work demonstrated that priming at the replication fork can occur through exogenously provided primers (11). These reactions require large excesses of oligonucleotides, presumably because the annealing reaction at the fork is kinetically driven.

**Figure 4.**
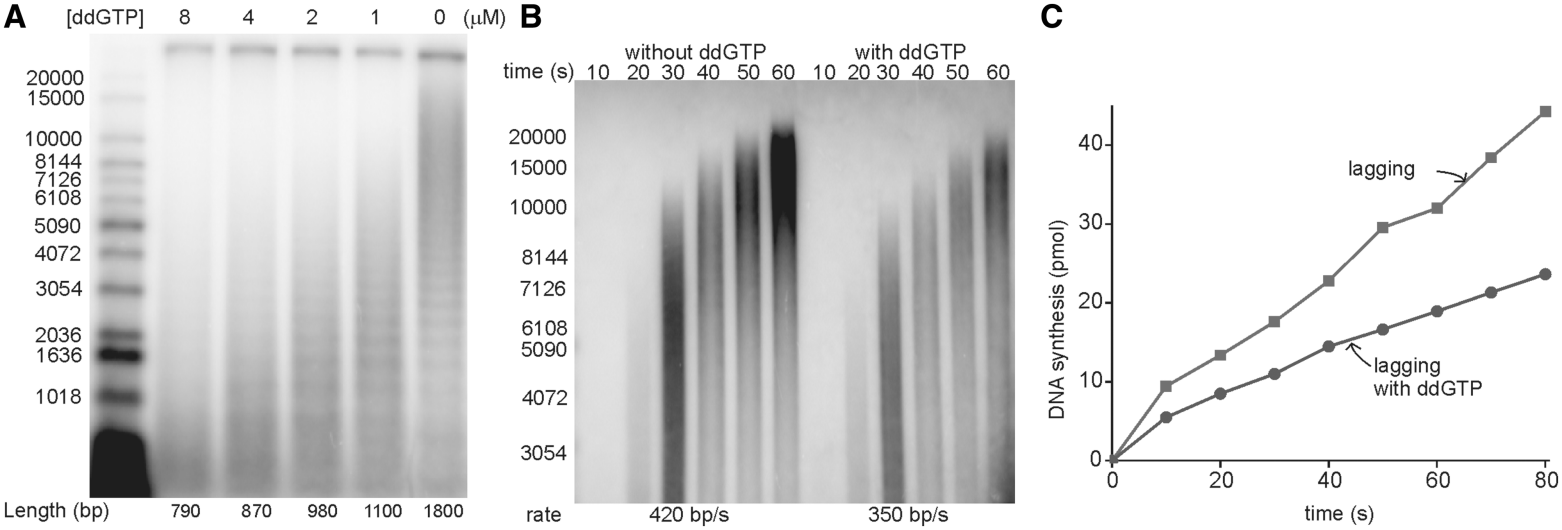
The availability of primers signals the lagging strand polymerase to cycle. **(A)** Optimized rolling circle reactions were carried out as described except 120 nM synthetic 15-mer primers substituted for primase and GTP, CTP and UTP. ddGTP was added to the designated concentrations at the same time radiolabeled nucleotide was added. Okazaki fragments incorporating α-[^32^P] dGTP (5000 cpm/pmol) were monitored by alkaline agarose gel electrophoresis. Lengths were determined with a 20-kb cutoff to exclude leading strand products. **(B)** In the presence or absence of 4 µM ddGTP, optimized rolling circle reactions were conducted as in (A) except α-[^32^P] dCTP was added at the same time with ATP and dNTPs. The 3 min elongation step in the presence of non-radioactive dNTPs before the addition of radioactive nucleotide was skipped so that the products would be short enough for accurate length quantification. Leading strand products incorporating α-[^32^P] dCTP (20 000 cpm/pmol) were monitored by alkaline agarose gel electrophoresis. The products of 20, 30 and 40 s were used to calculate the rate of leading strand synthesis. **(C)** The amount of lagging strand synthesis in the presence of 1 µM ddGTP was quantified.

We determined that, by omitting primase and substituting a 120-nM 15-mer complementary to a unique position within the 409 nt template, Okazaki fragments with an average length of ∼1800 nt could be obtained, comparable to the lengths observed if primers are synthesized by DnaG (Figure 4A). A banding pattern, separated by ∼400 nt, was consistent with priming occurring at a unique location (Figure 4A).

To determine if these exogenously provided primers could signal cycling, we titrated ddGTP into the reaction. The logic for this experiment was the same as explained for the experiment performed with DnaG-primed synthesis reported in Figure 2. If lagging strand cycling occurs through the collision model, synthesis should abort after initial Okazaki fragments incorporate ddGMP before encountering the 5′-end of the preceding Okazaki fragment. When increasing ddGTP is added to the reaction, Okazaki fragments become progressively smaller just as they do in the primase-primed reaction (Figure 4A). Comparison of the rates of leading strand synthesis in the presence and absence of ddGTP shows similar rates of synthesis indicating no significant perturbation of the rates of leading strand synthesis (Figure 4B). We observed robust synthesis of Okazaki fragments in the presence of synthetic primers, both in the presence and absence of ddGTP (Figure 4C). Okazaki fragment synthesis remains linear for over a minute in the presence of ddGTP.

## DISCUSSION

Two models have been proposed for providing the trigger to cause an otherwise highly processive replicase to rapidly cycle during Okazaki fragment synthesis. In the collision model, it had been posited that collision with the 5′-end of the preceding Okazaki fragment provided the signal. We recently eliminated the collision model by showing that it is kinetically incompetent—Pol III HE takes nearly 2 min to release and recycle upon collision with the 5′-end of model Okazaki fragments (24). The alternative signaling model proposed that synthesis of new primers by DnaG primase provided the signal for cycling (9).

To provide a rigorous test for the signaling model under conditions of balanced ongoing replication, we set up a large (409 nt) 5′-flapped minicircle system with highly asymmetric (50:1) G:C composition. This template not only allowed convenient monitoring of leading and lagging strand synthesis by quantifying radiolabeled dGMP or dCMP incorporation but also provided a means to selectively perturb lagging strand synthesis using dGTP anologs without affecting leading strand replication on its C-deficient template. In addition, as demonstrated in the bacteriophage T7 system (33,34), use of minicircles allows high template concentrations to be achieved so that substoichiometric helicase and replicase can be used, avoiding artifacts arising from action of these enzymes, if present in excess, on the initial replication products. Indeed, rolling circle replication reconstituted under these conditions behaved differently, showing a dependence on SSB and a dependence on PriA for leading strand replication.

As our first test of the signaling model, we prematurely terminated synthesis of lagging strands by adding increasing concentrations of the chain terminator ddGTP to reactions containing the four normal dNTPs. We observed shortening of Okazaki fragments because of incorporation of the ddGMP chain terminator. Yet, the rate of Okazaki fragment synthesis remained linear. This observation was only consistent with the signaling model whereby a stalled replicase on a prematurely terminated Okazaki fragment was induced to cycle to the next primer at the replication fork. An alternative explanation might have been that the Pol III HE stalled on a ddGMP terminated chain somehow had a decreased affinity and released more rapidly. That possibility was eliminated by direct experimental measurements of the rate of Pol III HE release from dNMP- and ddNMP-terminated chains (24). The rate of release of Pol III HE from a ddCMP terminated chain within a gap varies from 6 to 11 min, several 100-fold longer than the time required for synthesis of an Okazaki fragment.

To provide a further test of the signaling model, we substituted dGTP in reactions with dGDPNP. The chemistry step for insertion of this nucleotide is slowed and it also exhibits a higher *K*_m_ than dGTP. Thus, the rate of Okazaki fragment elongation can be ‘dialed in’ to the desired rate by decreasing dGDPNP concentrations. Using long single-stranded M13 templates, we were able to slow the elongation rate of Pol III HE from 570 nt/s (with 48 µM dGTP) to 23 nt/s (in the presence of 30 µM dGDPNP). The model M13 template contained ∼25% C. Our lagging strand minicircle template contained 45% C. Thus, we would expect the rate of lagging strand synthesis on the minicircle to be decreased even further, but direct measurements were not experimentally accessible. Under these conditions leading strand synthesis on the minicircle template was largely unaffected (420 nt/s). Thus, dGDPNP could be used to selectively slow the rate of lagging strand synthesis. We observed shortened Okazaki fragments in the presence of dGDPNP, consistent with the elongating lagging strand replicase being induced to release and recycle to the next primer synthesized at the replication fork before completion of Okazaki fragment synthesis.

If the collision mechanism was used to any significant level, even together with the signaling mechanism, it would take longer for an Okazaki fragment to be completed in the presence of dGDPNP. During this longer time, the replicase would have advanced, causing each Okazaki fragment to become increasingly longer. This outcome would be the opposite of our experimental observations. This provides additional evidence, in the context of a complete *E. coli* replication system, that the collision model is not operational. This result is consistent with our earlier studies on model lagging strands that showed the collision model was kinetically incompetent.

If Pol III HE is signaled to cycle prematurely because of slow elongation in the presence of dGDPNP, one would expect gaps between the resulting Okazaki fragments. We tested this prediction by elongation of the putative incomplete Okazaki fragments with a thermophilic polymerase that does not strand displace, a representation made by the manufacturer that we experimentally verified. All shortened Okazaki fragments, regardless of their length, were elongated to approximately the same length, consistent with a regular spacing of primers synthesized during rolling circle replication. We also experimentally verified that the rate of primer synthesis or utilization was not affected significantly by dGDPNP.

**Figure 5.**
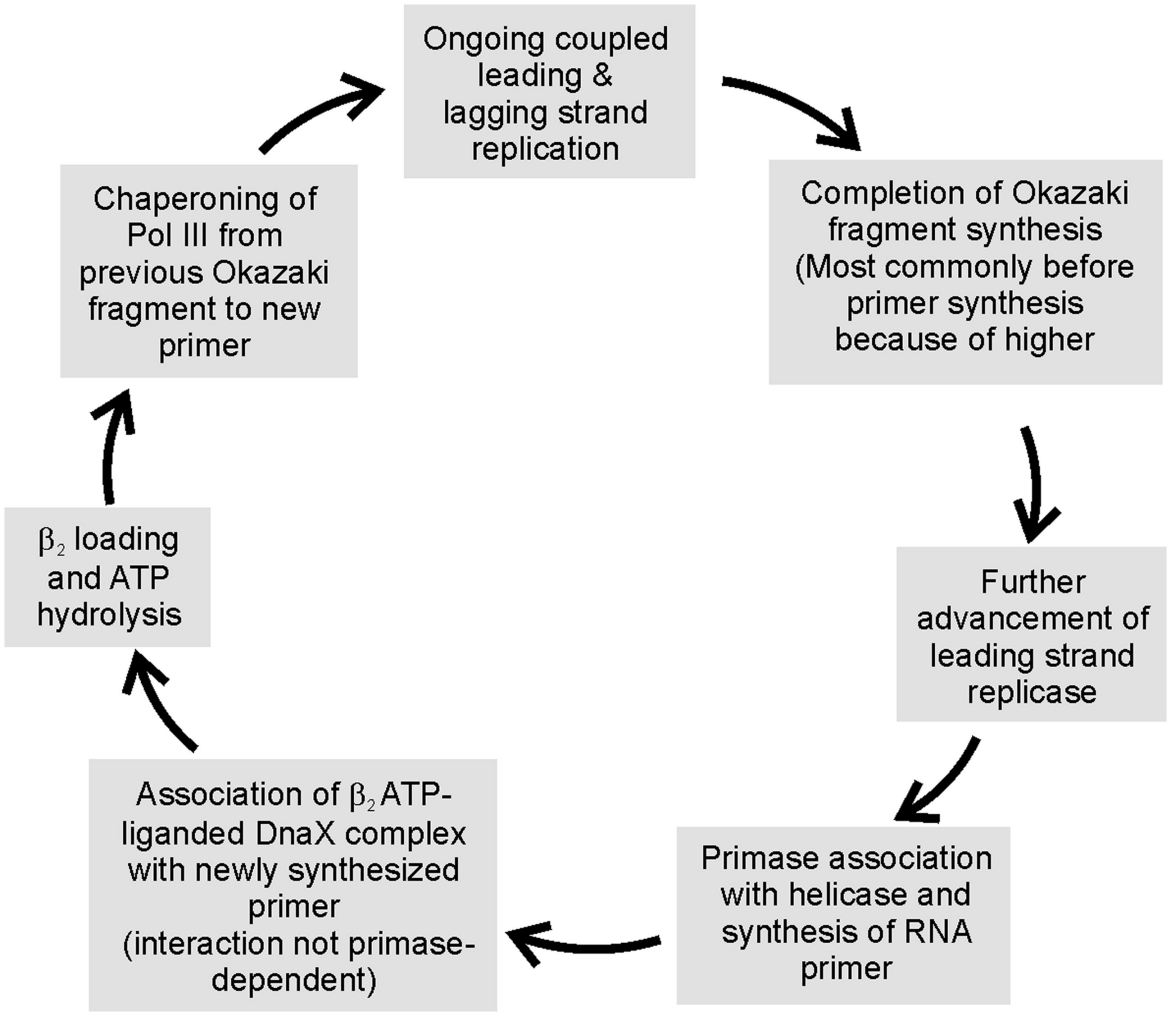
Proposed sequence of events in cycling of lagging strand polymerase during Okazaki fragment synthesis.

We note that Okazaki fragments synthesized in the presence of dGDPNP are not shortened to the same extent as would be predicted based on the slowing of replication on model M13 templates. In the presence of dGTP, Okazaki fragment length is only shortened 45% in the presence of 30 µM dGDPNP. Based on the difference in elongation rates on M13 templates, we would expect the length of Okazaki fragments to be shortened 25-fold. We have demonstrated that the rate of leading strand replication is not slowed. Hence, the rate of lagging strand synthesis using dGDPNP on coupled replication forks must be much faster than expected. Thus, the rate of polymerization is somehow accelerated significantly within the lagging strand polymerase, at least with dGDPNP. If the lagging strand polymerase rate is accelerated when using natural nucleotide precursors, that would provide an explanation of why gaps in Okazaki fragments are rarely observed, even though the collision model is not operational. If the lagging strand polymerase is much faster than the leading strand polymerase, it will complete its synthesis first and wait for the synthesis of a new primer before cycling (Figure 5). This model is consistent with whole cell single-molecule microscopy experiments. Using fluorescently tagged SSB, waves of occupancy at the replication fork have been observed (40). As initially pointed out, if the rates of leading and lagging strand replication were equivalent, SSB occupancy at the replication fork would remain constant. It was concluded from these studies that the rate of lagging strand polymerase elongation was significantly faster than leading strand elongation (40). Faster rates of lagging strand elongation have been proposed for bacteriophage T7 replication as well (41) and long ago were proposed as a theoretical feature of T4 DNA replication (42).

Having established that the signaling model and not the collision model operates at the replication fork, we sought to identify the signal. Clearly some event associated with synthesis of a new primer is involved, as proposed in the initial model (9). We sought to distinguish whether the signal emanated from primase (or its interaction with helicase or some other replisome component) or merely from the availability of a new primer. It has been previously demonstrated that high concentrations of exogenous synthetic primers can be used to drive Okazaki fragment synthesis (11). We applied the ddGTP technique used to demonstrate the use of the signaling model by the replisome with DnaG primase-synthesized primers to the replication system driven by exogenous primers and obtained the same result. Using a level of exogenous primer that gives approximately the same Okazaki fragment length as that obtained with primase, we titrated ddGTP and obtained ∼2-fold shortening with 4 µM ddGTP with both systems. Thus, it is just the presence of primers that provides the signal. The presence of primase is not required.

How might the signal be sensed? We have recently demonstrated that the DnaX complex not only loads β_2_ in an ATP-dependent reaction but also chaperones the associated polymerase onto the recently loaded β_2_ (43). Is it possible that the chaperoning reaction might be reversible with the ability to escort the polymerase off of an Okazaki fragment when appropriately signaled? When we found that Pol III HE took nearly 2 min to release when it collided with the preceding Okazaki fragment in a model system, we sought factors that might accelerate release. We found only one combination that did this: τ-containing DnaX complex, exogenous primed template and ATP (24). ATPγS could not substitute for ATP indicating ATP hydrolysis is probably required. β_2_ is not required to stimulate polymerase release but is presumably associated with DnaX under these conditions in cells, since its association is faster than the ATP-driven conformational change required for DNA binding (44). The acceleration observed was only 4-fold and the rates achieved were not adequate to support the kinetics of Okazaki fragment synthesis. However, when properly oriented by interaction with helicase and perhaps other components at the replication fork, DnaX complex might serve as a sensor. Thus, we incorporate DnaX complex as the sensor as a speculative feature of our cycling model (Figure 5). We note that the Benkovic laboratory observed that the concentration of clamp loader affected primer utilization and Okazaki fragment size at the T4 bacteriophage replication fork, raising the possibility that the T4 clamp loader might play a role in cycling of the lagging strand polymerase as well (25,45). Thus, the features of this evolving signaling model for triggering lagging strand replicase cycling during Okazaki fragment synthesis might be generally applicable.

## SUPPLEMENTARY DATA

Supplementary Data are available at NAR Online, including [46,47].

## FUNDING

National Science Foundation and the National Institutes of Health Training Grant [T-32 GM-065103]. Funding for open access charge: National Science Foundation.

*Conflict of interest statement.* None declared.

## Supplementary Material

Supplementary Data
