# Supplementary material for: Cycling of the E. coli lagging strand polymerase is triggered exclusively by the availability of a new primer at the replication fork
Source: Nucleic Acids Res. 2013 Nov 13;42(3):1747–56. doi: 10.1093/nar/gkt1098 (PMC3919610; doi:10.1093/nar/gkt1098)
Supplement: Supplementary Data [file supp_42_3_1747__index.html]

Cycling of the E. coli lagging strand polymerase is triggered exclusively by the availability of a new primer at the replication fork — Cycling of the E. coli lagging strand polymerase is triggered exclusively by the availability of a new primer at the replication fork — Supplementary Data 

# Cycling of the *E. coli* lagging strand polymerase is triggered exclusively by the availability of a new primer at the replication fork

## Supplementary Data

files

**Files in this Data Supplement:**

- Supplementary Data - pdf file
