# Supplementary material for: Cycling of the E. coli lagging strand polymerase is triggered exclusively by the availability of a new primer at the replication fork
Source: Nucleic Acids Res. 2013 Nov 13;42(3):1747–56. doi: 10.1093/nar/gkt1098 (PMC3919610; doi:10.1093/nar/gkt1098)
Supplement: Supplementary Data [file supp_gkt1098_nar-02463-h-2013-File007.pdf]

SUPPLEMENTAL DATA

CYCLING OF THE *E. COLI* LAGGING STRAND POLYMERASE IS TRIGGERED EXCLUSIVELY BY THE AVAILABILITY OF A NEW PRIMER AT THE REPLICATION FORK

Quan Yuan and Charles S. McHenry

Establishing a coupled rolling circle replication system on a minicircle template

In order to establish a coupled rolling circle replication system, all protein components required for *E. coli* rolling circle reactions were titrated in the presence of 1, 10, or 20 nM minicircle DNA templates (Supplementary Figure S1, S2, S3). Titration began with PriA, because it eliminated an unfavorable background originating from Pol III HE's strand displacement activity (32;36). First, a level of PriA was determined that blocked the strand displacement activity of Pol III HE in reactions performed in the absence of $DnaB_6$ (Supplementary Figure S1-3 A). When the selected level of PriA was used in the presence of $DnaB_{6,}$ partial inhibition was observed at lower template concentrations, presumably because of blocking a $DnaB_6$-independent background reaction (Supplementary Figure S1B, S2B). Then DnaG, $PriB_2$, $DnaT_3$, β_2, and SSB_4 were titrated and the optimal levels selected for further experiments (Supplementary Figure S1-3 C-G). Once a concentration of a component was selected, that concentration was used in subsequent titrations of other components. Since the optimal concentration of the DnaC helicase loader varies depending on the $DnaB_6$ concentration, DnaC was titrated against various concentrations of $DnaB_6$ (Supplementary Figure S1-3 H-J). In reactions containing 10 and 20 nM template, a substoichiometric level of $DnaB_6$ was selected for further experiments. Pol III* was titrated last (Supplementary Figure S1-3 K). Again, a substoichiometric level was selected for further experiments with 10 and 20 nM template. At the lowest (1 nM) template concentration, it was not possible to obtain adequate synthesis with substoichiometric helicase and Pol III*.

For 20 nM minicircle DNA template, the optimized concentrations of components were 0.5 μM SSB_4, 2.5 nM Pol III*, 100 nM β_2, 160 nM PriA, 50 nM $PriB_2$, 333 nM $DnaT_3$, 12 nM $DnaB_6$, 108 nM DnaC, and 25 nM DnaG (Supplementary Figure S3). For 10 nM minicircle DNA template, the optimized concentrations of components were 0.13 μM SSB_4, 1.25 nM Pol III*, 50 nM β_2, 65 nM PriA, 50 nM $PriB_2$, 333 nM $DnaT_3$, 6 nM $DnaB_6$, 100 nM DnaC, and 17 nM DnaG (Supplementary Figure S2). For 1 nM minicircle DNA template, 0.25 μM SSB_4, 2.5 nM Pol III*, 25 nM β_2, 20 nM PriA, 50 nM $PriB_2$, 333 nM $DnaT_3$, 6 nM $DnaB_6$, 72 nM DnaC, and 17 nM DnaG were selected. However, Pol III* and $DnaB_6$ were required in excess of a molar ratio to DNA to obtain adequate synthesis (Supplementary Figure S1).

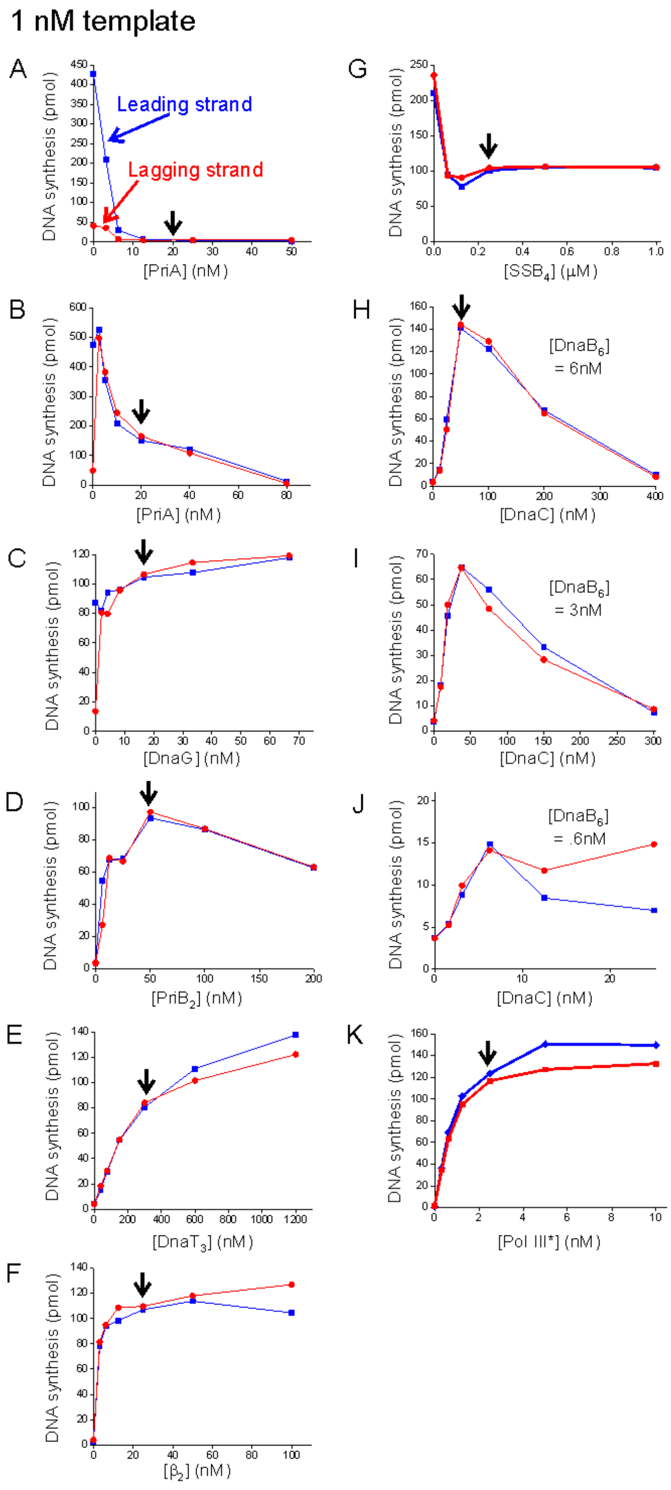

Figure S1. Protein requirements for *E. coli* rolling circle reactions on 1 nM minicircle DNA template, related to Figure 1. **(A)** Titration with PriA in the absence of $DnaB_6$. 0.25 μM SSB_4, 25 nM β_2, 2.5 nM Pol III*, 50 nM $PriB_2$, 300 nM $DnaT_3$, 100 nM DnaC, and 50 nM DnaG and varying amounts of PriA

were incubated with 1 nM mini-circle template, 0.2 mM UTP, 0.2 mM CTP, 0.2 mM GTP, 1.2 mM ATP, 100 μM dNTPs, and α-[^{32}P] dCTP or dGTP (2000 cpm/pmol) at 30°C in 25 μl. The reaction buffer was 10 mM magnesium acetate, 70 mM KCl, 50 mM Hepes (pH 7.5), 100 mM potassium glutamate, 20% glycerol, 200 μg/ml bovine serum albumin, 0.02% Nonidet P-40, and 10 mM dithiothreitol. The reaction was terminated by addition of EDTA to 20 mM final concentration after 19 min. Black arrows indicate the concentrations chosen for the subsequent titrations in all titrations. **(B)** Titration with PriA in the presence of $DnaB_6$. Reactions were conducted as in panel A except 6 nM $DnaB_6$ was added with the other protein components. **(C)** Titration with DnaG. Reactions were conducted as in panel B except 20 nM PriA was used. **(D)** Titration with $PriB_2$. Reactions were conducted as in panel C except 17 nM DnaG was used. **(E)** Titration with $DnaT_3$. Reactions were conducted as in panel D except 50 nM $PriB_2$ was used. **(F)** Titration with β_2. Reactions were conducted as in panel E except 333 nM $DnaT_3$ was used. **(G)** Titration with SSB_4. Reactions were conducted as in panel F except 25 nM β_2 was used.

(H) Titration with DnaC in the presence of 6 nM $DnaB_6$. Reactions were conducted as in panel G except 250 nM SSB_4 and 6 nM $DnaB_6$ were used. **(I)** Titration with DnaC in the presence of 3 nM $DnaB_6$. Reactions were conducted as in panel G except 250 nM SSB_4 and 3 nM $DnaB_6$ were used. **(J)** Titration with DnaC in the presence of 0.6 nM $DnaB_6$. Reactions were conducted as in panel G except 250 nM SSB_4 and 0.6 nM $DnaB_6$ were used. **(K)** Titration with Pol III*. Reactions were conducted as in panel H except 72 nM DnaC was used.

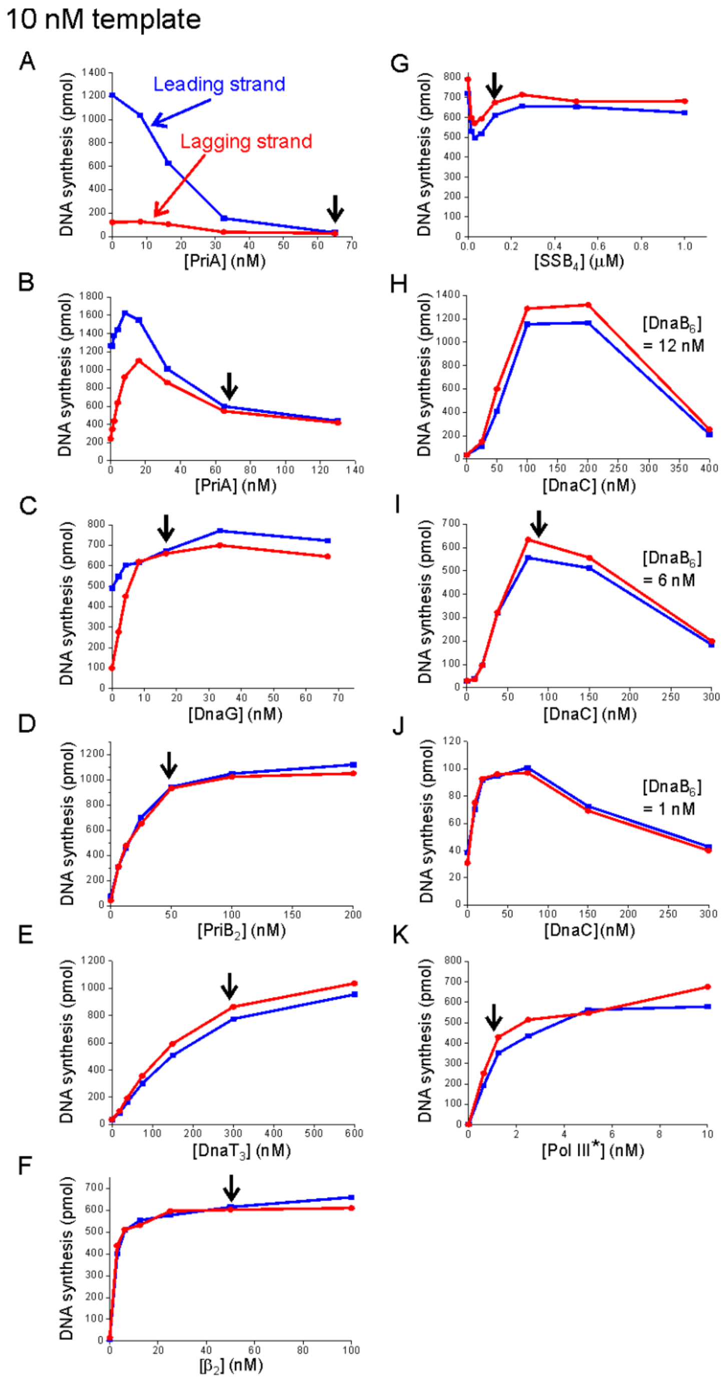

Figure S2. Protein Requirements for *E. coli* Rolling Circle Reactions on 10 nM Minicircle DNA Template, related to Figure 1. **(A)** Titration with PriA in the absence of $DnaB_6$. Reactions were conducted as described under the legend to Figure S1A except that the initial concentrations of protein components were 0.5 μM SSB_4, 50 nM β_2, 10 nM Pol III*, 50 nM $PriB_2$, 300 nM $DnaT_3$, 100 nM DnaC, and 33.3 nM DnaG, and reactions were terminated after 12 min. **(B)** Titration with PriA in the presence of 6 nM $DnaB_6$. Reactions were conducted as in panel A except 6 nM $DnaB_6$ was added with the other

protein components. **(C)** Titration with DnaG. Reactions were conducted as in panel B except 65 nM PriA was used. **(D)** Titration with $PriB_2$. Reactions were conducted as in panel C except 17 nM DnaG was used. **(E)** Titration with $DnaT_3$. Reactions were conducted as in panel D except 50 nM $PriB_2$ was used. **(F)** Titration with β_2. Reactions were conducted as in panel E except 333 nM $DnaT_3$ was used. **(G)** Titration with SSB_4. Reactions were conducted as in panel F except 50 nM β_2 was used. **(H)** Titration with DnaC in the presence of 12 nM $DnaB_6$. Reactions were conducted as in panel G except 130 nM SSB_4 and 12 nM $DnaB_6$ were used. **(I)** Titration with DnaC in the presence of 6 nM $DnaB_6$. Reactions were conducted as in panel G except 130 nM SSB_4 and 6 nM $DnaB_6$ were used. **(J)** Titration with DnaC in the presence of 1 nM $DnaB_6$. Reactions were conducted as in panel G except 130 nM SSB_4 and 1 nM $DnaB_6$ were used. **(K)** Titration with Pol III*. Reactions were conducted as in panel I except 100 nM DnaC was used.

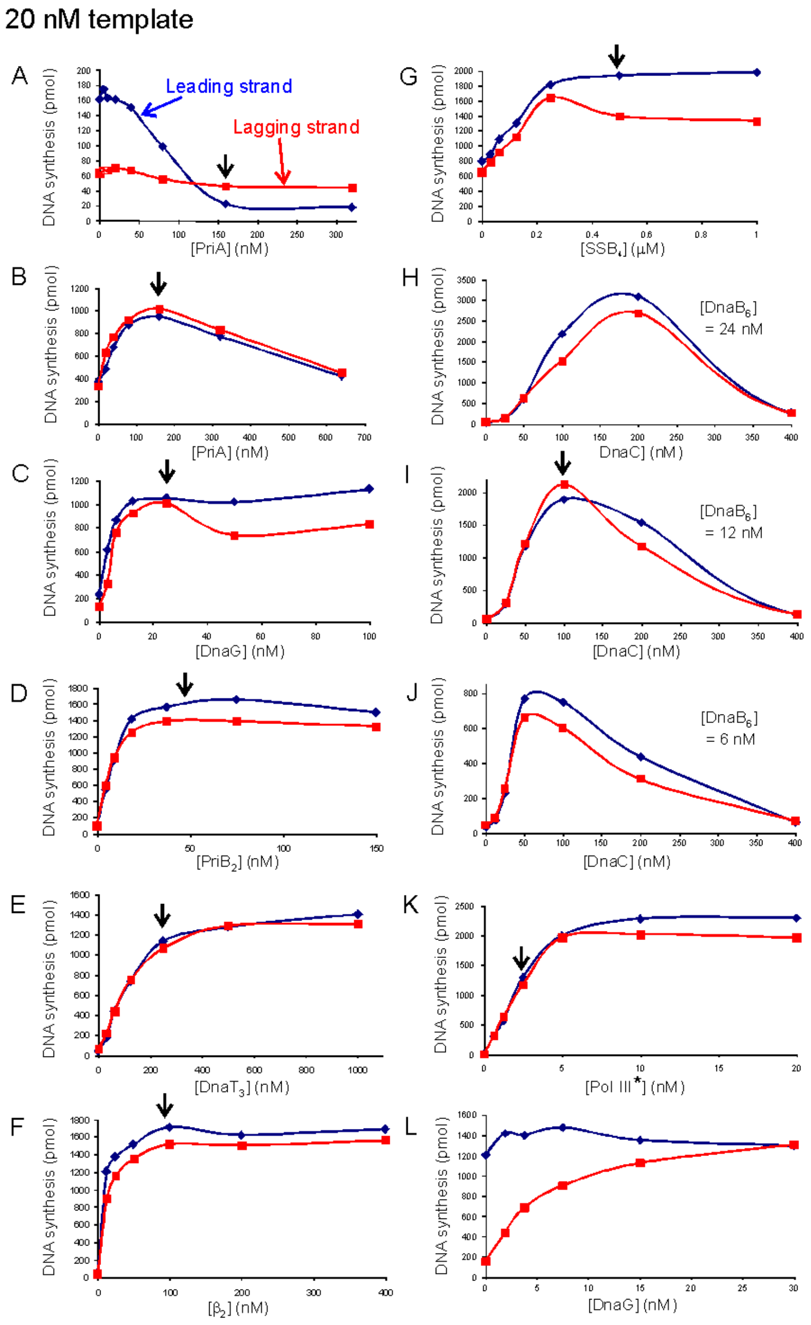

Figure S3. Protein requirements for *E. coli* rolling circle reactions on 20 nM minicircle DNA template, related to Figure 1. **(A)** Titration with PriA in the absence of $DnaB_6$. Reactions were conducted as described under the legend to Figure S1A except that the initial concentrations of protein components were 0.13 μM SSB_4, 50 nM β_2, 5 nM Pol III*, 50 nM $PriB_2$, 333 nM $DnaT_3$, 12 nM $DnaB_6$, 78 nM DnaC, and 50 nM DnaG, and reactions were terminated after 12 min. **(B)** Titration with PriA in the presence of 12 nM $DnaB_6$. Reactions were conducted as in panel A except 12 nM $DnaB_6$ was added with other protein components. **(C)** Titration with DnaG. Reactions were conducted as in panel B except 160 nM PriA was

used. **(D)** Titration with $PriB_2$. Reactions were conducted as in panel C except 25 nM DnaG was used. **(E)** Titration with $DnaT_3$. Reactions were conducted as in panel D except 50 nM $PriB_2$ was used. **(F)** Titration with β_2. Reactions were conducted as in panel E except 333 nM $DnaT_3$ was used. **(G)** Titration with SSB_4. Reactions were conducted as in panel F except 100 nM β_2 was used. **(H)** Titration with DnaC in the presence of 24 nM $DnaB_6$. Reactions were conducted as in panel G except 500 nM SSB_4 and 24 nM $DnaB_6$ were used. **(I)** Titration with DnaC in the presence of 12 nM $DnaB_6$. Reactions were conducted as in panel G except 500 nM SSB_4 and 12 nM $DnaB_6$ were used. **(J)** Titration with DnaC in the presence of 6 nM $DnaB_6$. Reactions were conducted as in panel G except 500 nM SSB_4 and 6 nM $DnaB_6$ were used. **(K)** Titration with Pol III*. Reactions were conducted as in panel I except 108 nM DnaC was used. **(L)** Titration with DnaG. Reactions were conducted as in panel K except 2.5 nM Pol III* was used. Note that the dependency on DnaG for leading strand synthesis observed under suboptimal conditions (panel C) is no longer observed under the fully optimized conditions represented in this panel.

Preincubation of reaction components minimizes the lag phase and permits synchronization of the rolling circle reaction

Simply mixing all components together leads to a rolling circle reaction with a lag phase at the beginning due to unsynchronized DNA synthesis. To minimize this problem, a pre-initiation complex of all protein components was assembled on the template in the presence of ATPγS, CTP, GTP, and UTP. Omitting ATP from the pre-incubation stage ensures that helicase can be loaded but not translocated. Once dNTPs and ATP were added, synthesis of each strand initiates with a reduced lag phase (Figure S4). A final concentration of 5 μM ATPγS was required for reactions containing 20 nM and 10 nM template with pre-incubation times of 5 and 11 min, respectively. A final concentration of 10 μM ATPγS was required for reactions containing 1 nM template with a 17 min pre-incubation time.

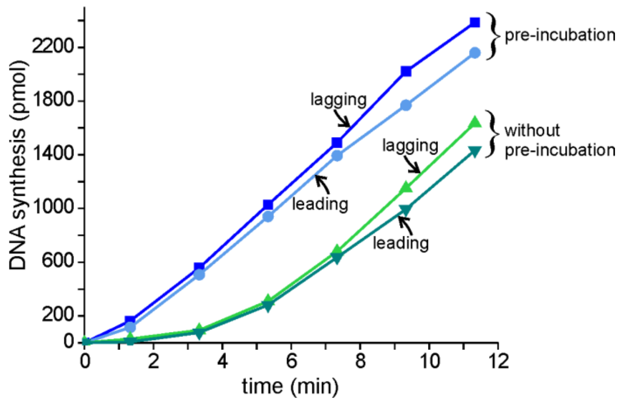

Figure S4. Preincubation of reaction components in the presence of ATPγS supports formation of a preinitiation complex and elimination of a lag phase, related to Figure 2. The data shown were obtained using 20 nM template.

Increasing concentrations of ddGTP result in decreased levels of radioactive nucleotide incorporation in the lagging strand, but permit maintenance of linear synthesis rates

As the concentration of ddGTP is increased beyond the level shown in Figure 2C in the main article, the level of incorporation of dNTPs in the lagging strand product decreases (Figure S5). This is, in part, the result of the synthesis of shorter Okazaki fragments (Figure 2A). But, there is a reduction of the overall molar level of Okazaki fragment synthesis, indicating some level of perturbation. Nevertheless, the rate of Okazaki fragment synthesis remains linear for over two minutes, even in the presence of the highest ddGTP concentrations.

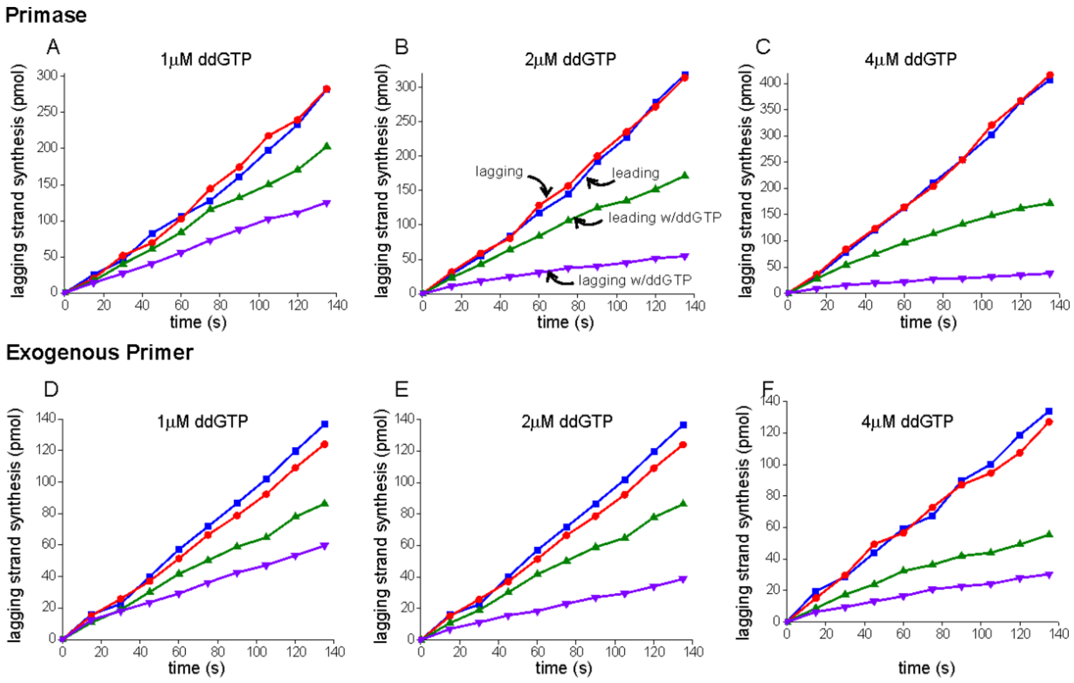

Figure S5. A linear rate of Okazaki fragment synthesis is maintained as the concentration of ddGTP increases, related to Figure 4. **(A-C)** Reactions were performed under conditions of the optimized rolling circle reaction with 1, 2, and 4 µM ddGTP added at the same time as radiolabeled nucleotide. The amount of leading and lagging strand synthesis in the absence (leading-blue; lagging-red) and presence (leading-green; lagging-purple) of ddGTP was quantified. **(D-F)** The same experiments as A-C were performed except 120 nM exogenous synthetic 15-mer primers (TGATAGGGGGTATGG) replaced primase, GTP, UTP and CTP.

dGDPNP displays a higher apparent K_m for the DNA polymerase III holoenzyme-catalyzed reaction and can be used to modulate the elongation rate

The DNA Pol III HE incorporates dGDPNP with a 20-fold higher apparent K_m than dGTP (Figure S6A,B). The K_m for dGDPNP (40 μM) is sufficiently high that reactions can be conducted at sub-K_m concentrations without danger of depleting nucleotides. This allowed modulation of the rate of elongation on cytosine-containing templates and allowed us to selectively slow the rate of lagging strand synthesis when templates with an asymmetric G:C distribution were used. The measured K_m is an estimate and expressed as an apparent value, because the reaction kinetics monitored included both initiation complex formation and elongation stages.

On a template containing approximately 25% C, the rate of elongation in the presence of near-saturating dGDPNP was reduced to 57 nt/s, 10-fold slower than the 570 nt/s measured in the presence of saturating dGTP (Figure S6C). Thus, dGDPNP may also be slowing the chemistry step of the reaction sufficiently that it becomes rate-limiting. Decreasing dGDPNP to a level of 0.75 K_m decreased the observed elongation rate about 25-fold relative to dGTP. In contrast, the rate of leading strand elongation on a minicircle template containing only two G residues is not slowed significantly (Figure S6D).

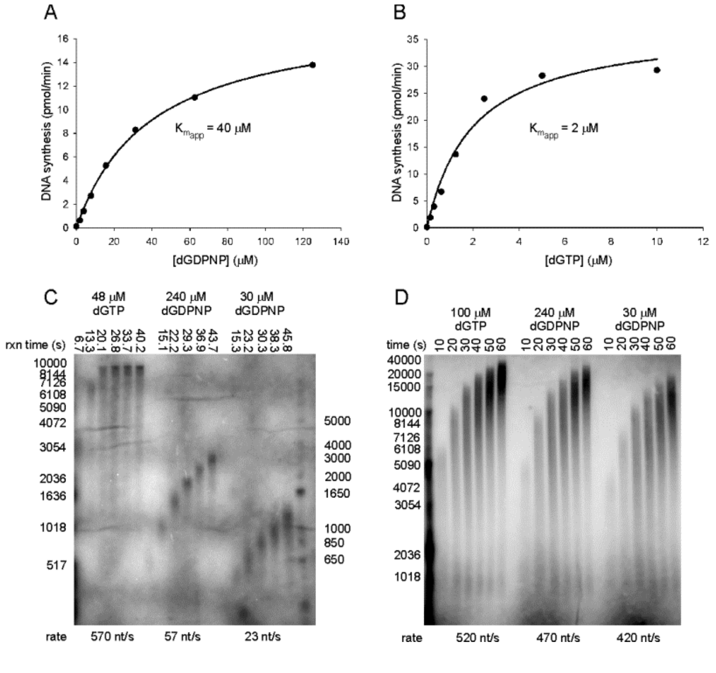

Figure S6. dGDPNP can be used to modulate the rate of elongation on cytosine-containing templates, related to Figure 3. **(A,B)** The apparent K_m values for dGTP and dGDPNP are 40 μM and 2 μM, respectively. Reactions containing 0.6 μM SSB_4, 15 nM β_2, 10 nM Pol III*, and 2.3 nM primed M13Gori

DNA (38), 100 μM ATP, 18 μM dTTP, 48 μM dATP, 48 μM dCTP, ^{3}H-dTTP (760 cpm/pmol) and varying amounts of dGTP or dGDPNP were incubated at 30°C for 2 min (dGTP) or 5 min (dGDPNP). dNTP incorporation was determined by scintillation counting. The titration curves were fit to the Michaelis-Menten equation, $V=V_{max}[S]/(K_m+[S])$, by SigmaPlot's nonlinear regression tool. [S] was the concentration of dGTPs or dGDPNPs, and *V* was the rate of dNTP incorporation. **(C**) Elongation rates on the primed M13Gori DNA template in the presence of indicated amounts of dGTP or dGDPNP. Reactions were monitored on a 0.9% alkaline agarose gel. Products with dGTP at 6.7 s, 13.3 s, and 20.1 s and products with dGDPNP at all time points were used to determine the elongation rate. **(D)** Rate of leading strand synthesis at 20 mM minicircle template in the presence of the indicated amounts of dGTP or dGDPNP. The time course of the reaction was monitored on a 0.5% alkaline agarose gel, and products taken at 10 s, 20 s, and 30 s were used to determine the rate of leading strand synthesis.

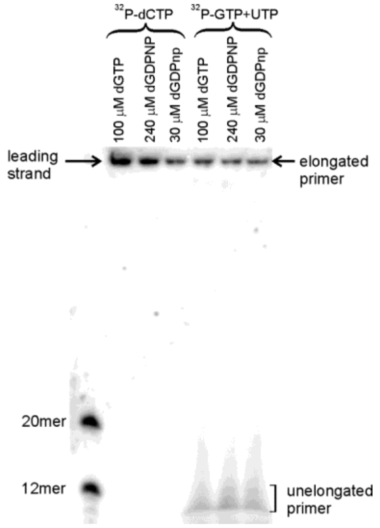

Figure S7. dGDPNP does not perturb the frequency of primer synthesis or primer utilization, related to Table 1. Two identical sets of optimized rolling circle reactions with 20 mM templates were carried out in the presence of dGTP or dGDPNP, except the reactions were stopped at 10 min after the addition of α-[^{32}P] dCTP or dGTP. α-[^{32}P] dCTP (400 cpm/pmol) was employed in the left set to reveal leading strand products, and α-[^{32}P] GTP and UTP (12,000 cpm/pmol) in the right set to permit detection of RNA primers.

SUPPLEMENTAL EXPERIMENTAL PROCEDURES

Buffers

All reactions on minicircle templates were conducted with buffer containing 10 mM magnesium acetate, 70 mM NaCl, 50 mM HEPES (pH 7.5), 100 mM potassium glutamate, 20% glycerol, 200 μg/ml bovine serum albumin, 0.02% Nonidet P (NP) -40, and 10 mM dithiothreitol (DTT). Buffer T contains 50 mM Tris (pH 7.5), 10% glycerol, 0.5 mM EDTA, and 5 mM DTT. Buffer B contains 50 mM Tris (pH 8), 20% glycerol, 1 mM EDTA, 5 mM DTT, and 0.01% NP-40. Buffer C contains 50 mM Tris (pH 8), 10% glycerol, 1 mM EDTA, 5 mM DTT, 0.01% NP-40, and 50 mM NaCl. Buffer A contains 50 mM Tris (pH 7.5), 20% glycerol, and 5 mM DTT. Buffer F contains 50 mM HEPES (pH 7.5), 100 mM NaCl, 10% glycerol, 0.25 mM EDTA, 0.01% NP-40, and 5 mM DTT. The stop mix used for stopping DNA replication reactions contained 40 mM Tris (pH 8), 0.2% SDS, 100 mM EDTA, and 50 μg/ml proteinase K.

Minicircle DNA template preparation

A fragment containing linear minicircle DNA was amplified by running 4000 x 100 μl PCR reactions containing 100 ng of plasmid pBsRC3 DNA (37), 2 μM each of forward primer (5'Biotin-TGT GGA ATT GTG AGC GGA TA) and reverse primer (5'Biotin-GTT TTC CCA GTC ACG ACG TT), 200 μM dNTPs, and 2.4 U of *Pfu* polymerase. (One unit is defined as the amount of enzyme required to catalyze the incorporation of 10 nmol of dNTPs into acid-insoluble material in 30 min at 75°C). The PCR reaction was performed at 94°C for 3 min, followed by 30 cycles at 94°C for 25 s, 60°C for 15 s, and 72°C for 30 s, and an extra 10 min at 72°C at the end. A 1.5% agarose gel showed that more than 95% amplified products were the target DNA (Figure S8B lane 1, Figure S8A step 1). The PCR product (80 mg) was extracted with one volume of phenol/chloroform/isoamyl alcohol (25:24:1) and one volume of chloroform, and precipitated by the addition of 0.5 volumes of 5 M ammonium acetate and 1.5 volumes of isopropyl alcohol. The pellet was washed with 70% ethanol and dissolved in 10 mM Tris-HCl buffer (pH 8) to 1 μg/μl. The purified PCR product (40 mg) was digested with EcoRI (666 U/mg DNA) at 37°C for 9 h, and the digestion was stopped by heating at 65°C for 40 min. Digestion was >90% completed (Figure S8B lane 2, Figure S8A step 2a). The linear minicircle DNA was separated from biotin-containing terminal fragments created by EcoRI digestion by passing the digested DNA over a high capacity streptavidin resin (Pierce, 10 ml). Electrophoresis in 1.5% agarose showed that more than 95% of the product was the linear minicircle DNA (Figure S8C lane 2, Figure S8A step 2b). The purified linear DNA (27 mg) was diluted to 2.5 μg/ml and ligated using ligase (0.4 U/ml DNA solution, Epicentre)

in 33 mM Tris-acetate (pH 7.8), 66 mM potassium acetate, 10 mM magnesium acetate, 5 mM DTT, and 1 mM ATP at 16°C for 20 h (Figure S8D lane 2, S8A step 3a). The unligated fragment and linear multi-ligated product were digested completely from both 3' and 5' ends by the combination of lambda exonuclease (0.3 U/ml ligation mixture), exonuclease I (0.3 U/ml ligation mixture), and exonuclease III (1 U/ml ligation mixture) at 37°C for 8 h. All enzymes were thermally inactivated at 80°C for 20 min. Electrophoresis in 2% agarose showed that more than 90% of the product was the ligated minicircle DNA (Figure S8D lane 3, Figure S8A step 3b). NaCl was added to the ligation reaction mixture to 0.5 M final concentration. Then the ligated minicircle DNA was loaded onto three QIAGEN-tip 10000 columns. The column was washed with Qiagen Buffer QC and the DNA eluted with Qiagen Buffer QF. DNA was precipitated by adding 0.7 volumes of isopropanol, washed with 70% ethanol, and dissolved in TE buffer to 1 μg/μl (Figure S8A step 3c). The purified product (7 mg) was nicked at the single recognition site with Nt. BstNBI nicking enzyme (2 U/μg DNA) at 55°C for 3 h. Nt. BstNBI was thermally inactivated at 80°C for 30 min (Figure S8A step 4). The nicked DNA was incubated with Vent polymerase (0.75 U/μg DNA) and 300 μM dATP, dCTP, and dTTP at 75°C for 2 h to form a 394-bp-long DNA flap (37). EDTA was added to a final concentration of 25 mM. The tailed DNA was purified by phenol chloroform extraction and isopropyl alcohol purification as described above. Electrophoresis in 2% agarose indicated a yield >80% (Figure S8E lane 2, Figure S8A step 5).

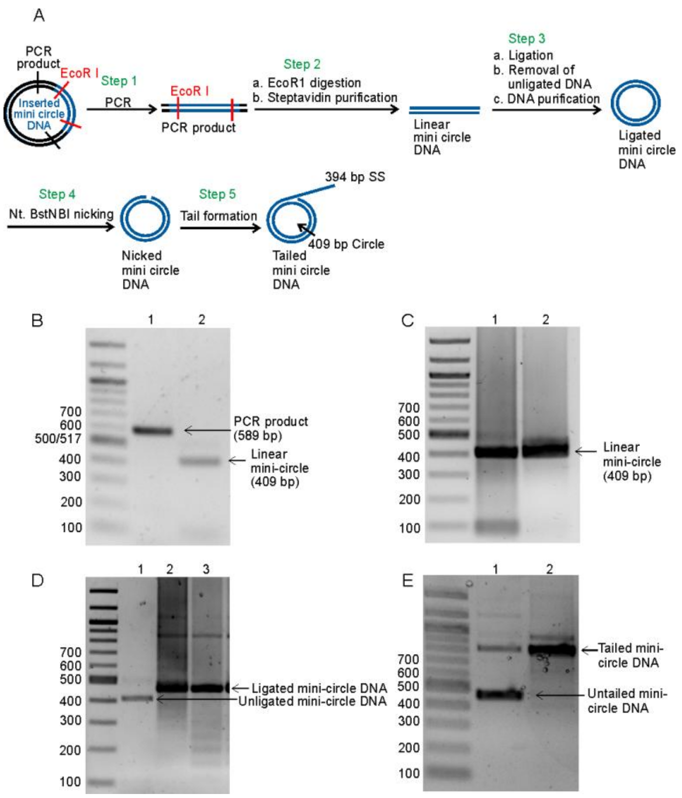

Figure S8. Preparation of minicircle DNA template. **(A)** General scheme for template preparation. Important Intermediate Products and Final Product of Minicircle Template Preparation: **(B)** Lane 1: The PCR product (589 bp); Lane 2: EcoRI digested products. **(C)** Lane 1: EcoRI digested products; Lane 2: Linear minicircle fragment (409 bp) after streptavidin purification. **(D)** Lane 1: Purified unligated linear minicircle fragment; Lane 2: Ligated minicircle DNA; Lane 3: Ligated minicircle DNA after exonuclease digestion of linear fragments. (**E**) Lane 1: Purified untailed circular minicircle fragment; Lane 2: Tailed minicircle DNA template after purification.

Protein purifications

Cells were grown in a 250 l fermentor in F-medium (31) supplemented with 1% glucose at 37°C in the presence of ampicillin (100 mg/l for PriA, DnaT and Pol III*, 200 mg/l for PriB, 50 mg/l for DnaC) and chloramphenicol (25 mg/l for PriA, PriB, and DnaT, 10 mg/l for DnaC). When OD_{600}=0.5-0.6, IPTG was added to 1 mM. Another 100 mg/l ampicillin was added at induction for PriA and DnaT, and 200 mg/l ampicillin was added at induction and 1 h post-induction for PriB. For Pol III*, when OD_{600}=0.83, 200 mg/l ampicillin was added and additional 200 mg/l ampicillin was added at 2 h post-induction. Cells expressing PriA and PriB were harvested after 2 h of induction, and cells expressing DnaT, DnaC, and

Pol III* after 3 h of induction. Harvested cells were lysed to form fraction (Fr) I (46). Proteins were precipitated with indicated concentrations of ammonium sulfate and centrifuged at 23,000 g for 60 min. The resuspended pellet was Fr II.

PriA and other primosomal proteins were performed by modifications of published procedures (27). Fr II was prepared by addition of ammonium sulfate to 50% saturation to Fr I (generated from 50 g cells). The pellet was resuspended in Buffer T to a conductivity equivalent to that of Buffer T+100 mM NaCl. The solution was loaded onto an SP Sepharose column (80 ml) equilibrated with Buffer T+100 mM NaCl. The column was washed with 6 volumes of the same buffer, and proteins were eluted with 10 volumes of Buffer T with a 100 mM-800 mM NaCl gradient. PriA eluted with Buffer T+400 mM and was pooled and precipitated by addition of solid ammonium sulfate to 50% saturation. The pellet was resuspended in 4 ml of Buffer T+150 mM NaCl and 20% glycerol, and loaded onto a Sephacryl 200 column (130 ml) equilibrated with Buffer T+150 mM NaCl. The eluate containing PriA (16 mg) was collected, aliquoted, frozen in liquid N_2, and stored at -80°C.

PriB Fr II was prepared by addition of ammonium sulfate to 50% saturation to Fr I (generated from 40 g cells). The pellet was resuspended in Buffer T and the conductivity of the resulting solution was adjusted to that of Buffer T+200 mM NaCl. The resulting solution was loaded onto an SP Sepharose column (80 ml) equilibrated with Buffer T+100 mM NaCl. The column was washed with 5 volumes of Buffer T+200 mM NaCl, and proteins were eluted with 12.5 volumes of Buffer T with a 200 mM-700 mM NaCl gradient. PriB eluted with Buffer T+330 mM NaCl and was pooled and loaded onto a Heparin Sepharose column (40 ml) equilibrated with Buffer T+200 mM NaCl. The column was washed with 5 volumes of the same buffer, and proteins were eluted with 10 volumes of Buffer T with a 200 mM-700 mM NaCl gradient. PriB eluted in Buffer T+420 mM NaCl and was pooled, diluted with Buffer T to make the conductivity equivalent to that of Buffer T+100 mM NaCl, and loaded onto a Hi-TRAP SP Sepharose XL column (5 ml) as a concentration step. Proteins were step eluted with Buffer T+700 mM NaCl. The concentrated proteins were loaded onto a Hi-Load 16/60 Superdex 200 column equilibrated with Buffer T+300 mM NaCl. The eluate containing PriB (40 mg) was collected, aliquoted, frozen in liquid N_2, and stored at -80°C.

DnaT Fr II was prepared by addition of ammonium sulfate to 50% saturation to Fr I (generated from 150 g cells). The pellet was resuspended in Buffer T and the conductivity of the resulting solution was adjusted to that of Buffer T+100 mM NaCl. The resulting solution was loaded onto a Q Sepharose column (70 ml) equilibrated with Buffer T+100 mM NaCl. The column was washed with 6 volumes of Buffer T+100 mM NaCl, and proteins were eluted with 14 volumes of Buffer T with a 100 mM-450 mM

NaCl gradient. DnaT eluted in Buffer T+220 mM NaCl and was pooled and loaded onto a Heparin Sepharose column (110 ml) equilibrated with Buffer T+100 mM NaCl. The column was washed with 7 volumes of Buffer T+100 mM NaCl, and proteins were eluted with 12 volumes of Buffer T with a 100 mM-500 mM NaCl gradient. DnaT eluted with Buffer T+280 mM NaCl was pooled and precipitated by addition of ammonium sulfate to 65% saturation. The pellet was resuspended by 2 ml of Buffer T+150 mM NaCl and 30% glycerol, and loaded onto a Sephacryl 200 column (105 ml) equilibrated with Buffer T+150mM NaCl and 30% glycerol. The eluate containing DnaT (35 mg) was collected, aliquoted, frozen in liquid N_2, and stored at -80°C.

DnaC Fr II was prepared by addition of 0.075% polyethyleneimine and 50% saturated ammonium sulfate to Fr I (generated from 110 g cells). The pellet was resuspended in Buffer B and the conductivity of the resulting solution was similar to that of Buffer B+20 mM NaCl. The resulting solution was loaded onto a Q Sepharose column (210 ml) equilibrated with Buffer B+20 mM NaCl. The column was washed with 3 volumes of Buffer B+20 mM NaCl, and the flow-through was collected and loaded onto a phosphocellulose column (40 ml) equilibrated with Buffer B+20 mM NaCl. The column was washed with 3 volumes of Buffer B+20 mM NaCl, and proteins were eluted with 10 volumes of Buffer B with a 20 mM-300 mM NaCl gradient. DnaC eluted with Buffer B+180 mM NaCl was pooled and loaded onto a hydroxyapatite column (11 ml) equilibrated with Buffer B+50 mM NaCl. The column was washed with 6 volumes of Buffer B+50 mM NaCl, and proteins were eluted with 15 volumes of Buffer C with a 0 mM-300 mM ammonium sulfate gradient. DnaC eluted with Buffer C+90 mM ammonium sulfate was pooled and dialyzed against Buffer B+150mM NaCl and 30% glycerol. Purified DnaC (10 mg) was collected, aliquoted, frozen in liquid N_2, and stored at -80°C.

To prepare Pol III* Fr II, Fr I (generated from 125 g cells) was adjusted to 40% ammonium sulfate. Contaminants were removed by backwashing with decreasing amounts of ammonium sulfate first using a 0.20 ammonium sulfate backwash (specified as g added to each ml buffer), followed by a 0.17 ammonium sulfate backwash to generate Fr II as described (46). The pellet (147 mg protein; $8.6x10^7$ units) was resuspended in Buffer A and the conductivity of the resulting solution was adjusted to that of Buffer A+20 mM NaCl. The resulting solution was loaded onto an SP Sepharose column (100 ml) equilibrated with Buffer A+20 mM NaCl. The column was washed with 3 volumes of Buffer A+20 mM NaCl, and proteins were eluted with 10 volumes of Buffer A with a 20 mM-200 mM NaCl gradient. Pol III* eluted with Buffer A+110 mM NaCl and was pooled and precipitated by addition of ammonium sulfate to 55% saturation. The pellet was resuspended by 0.85 ml of Buffer F (5.1 mg protein; $1.5x10^7$ units), and loaded onto a Sephacryl S-300 column (10 ml) equilibrated with Buffer F. The eluate

containing Pol III* (3.8 mg, 1.1x10^7 units) was collected, aliquoted, frozen in liquid N_2, and stored at -80°C.

Concentrations of all proteins were measured with a Bradford protein assay using the Albumin Standard from Pierce (47).

Alkaline agarose gel electrophoresis

For the analysis of the size of lagging strand products, samples were digested with protease K (30 min, 37°C, 25 μg/ml); mixed with 30 mM NaOH, 2 mM EDTA, 2% glycerol, and 0.02% bromophenol blue; and fractionated on 0.6% alkaline agarose gels for approximately 18 h at 24 V in a running buffer of 30 mM NaOH and 2 mM EDTA. Gels were fixed in 8% (w/v) trichloroacetic acid, dried onto DEAE paper, autoradiographed on storage phosphor screens, and scanned with a PhosphorImager.

Development of a method to fill gaps between Okazaki fragments without strand displacement of the downstream Okazaki fragment

Gaps between incomplete Okazaki fragments were filled by thermophilic polymerases to minimize issues that might result from secondary structure within gaps. To obtain accurate quantification of gap size, we needed to ensure that the polymerase used did not catalyze strand displacement synthesis into the downstream Okazaki fragment. Under the reaction conditions we employed, Pfu catalyzed an unacceptable level of strand displacement synthesis. We pursued additional polymerases and tried Phusion because the supplier (New England Biolabs) indicated it did not strand displace. We observed moderate strand displacement (<150 nt) above one unit of polymerase per 20 μl reaction, but not at lower levels (Figure S9). We thus used 0.2 U of Phusion for gap filling of purified products resulting from rolling circle replication.

After the rolling circle product from each 25 μl reaction was extracted with phenol-chloroform and precipitated with isopropanol, it was incubated with 100 μM dNTPs, 0.2 U Phusion polymerase, and ^{32}P-dATP (2 μCi/reaction) at 72°C for 15 min. Gap filling products with 0.2 U and 1 U of Phusion were compared side by side, and no difference was found. According to the supplier, Phusion extends DNA at a rate of 15-30 s/kb, which is 10 times faster than Pfu. Since 30 min was chosen for gap filling reactions with Pfu (25), 15 min should be long enough to fill all gaps with Phusion.

Normalizing the lengths of gap-filled Okazaki fragments requires the same specific activity of the radioactive nucleotides as in the original Okazaki fragments with gaps between them. Without addition of radioactivity in the gap filling reaction, the lengths might be biased towards the lower molecular weight. However, we also need to make sure that not too many unused primers are elongated during gap filling, which may obscure the true size of gaps. Therefore, a comparison of gap filling was made in the

presence and absence of the same amount of ^{32}P-dATP as the rolling circle reaction. No significantly different lengths in two conditions were found, which relieved our concerns (Figure S9).

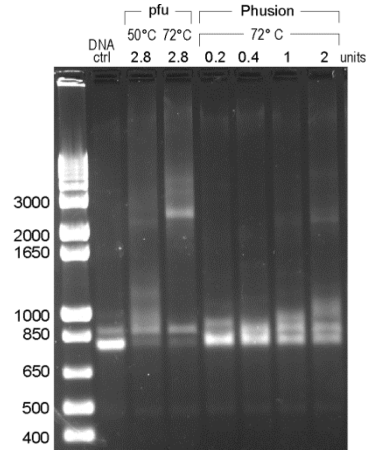

Figure S9. Phusion polymerase does not strand-displace under conditions used for determination of gap size between Okazaki fragments. Strand displacement activity of Pfu and Phusion polymerase (New England Biolabs). We incubated 28 nM minicircle template, 250 μM dNTPs, and the indicated amounts of Pfu or Phusion polymerase (20 μl, 30 min) at the indicated temperatures. The 'DNA ctrl' lane contains the 800 bp long minicircle template without any polymerase. Products were monitored on an ethidium bromide stained 2% agarose gel. (1 unit = 10 nmol dNTP incorporation/ 30 min.)

Determination of primer utilization and the amounts of GMP+UMP per Okazaki fragment

Rolling circle reactions were carried out in the presence of α-[^{32}P] GTP and α-[^{32}P] UTP (specific activity 12,000 cpm/pmol) or α-[^{32}P] dCTP (specific activity 400 cpm/pmol). The sample was loaded onto a 20% denaturing polyacrylamide gel in 50% w/v urea. γ-[^{32}P] ATP labeled 12-mer RNA and 20-mer DNA were loaded as markers. The gel was prerun at 14 W for 30 min and then run at 12 W for 3.5 h. The gel was dried directly with no pre-treatment on DEAE paper, exposed to a phosphorimage screen for 18 hours, and scanned with a PhosphorImager. DNA fragments of 10-14 nt in length were quantified as free primers, and the dark bands at the top of the gel were quantified as elongated primers.

The quantification from scintillation counting was directly proportional to the pixel density from phosphorimaging. Thus, 1 μl of α-[^{32}P] G/UTP and 1 μl of α-[^{32}P] dCTP were spotted on a GFC filter paper and the counts were determined with a scintillation counter. The specific activity of α-[^{32}P] G/UTP and α-[^{32}P] dCTP were calculated by the count divided by the amount of each nucleotide in a reaction. A

relative amount of α-[^{32}P] G/UTP and α-[^{32}P] dCTP incorporation was calculated by the pixel density of bands divided by the specific activity of the corresponding radioactive material. This term was directly comparable among different bands.

SUPPLEMENTAL REFERENCES

25. Yang,J., Nelson,S.W. and Benkovic,S.J. (2006) The control mechanism for lagging strand polymerase recycling during bacteriophage T4 DNA replication. *Mol. Cell*, **21**, 153-164.
27. Marians,K.J. (1995) phi X174-Type Primosomal Proteins: Purification and Assay. *Methods Enzymol.*, **262**, 507-521.
31. Kim,D.R. and McHenry,C.S. (1996) Biotin tagging deletion analysis of domain limits involved in protein-macromolecular interactions: Mapping the τ binding domain of the DNA polymerase III α subunit. *J. Biol. Chem.*, **271**, 20690-20698.
32. Yuan,Q. and McHenry,C.S. (2009) Strand displacement by DNA polymerase III occurs through a τ-ψ-χ link to SSB coating the lagging strand template. *J. Biol. Chem.*, **284**, 31672-31679.
36. Xu,L. and Marians,K.J. (2003) PriA mediates DNA replication pathway choice at recombination intermediates. *Mol. Cell*, **11**, 817-826.
37. Sanders,G.M., Dallmann,H.G. and McHenry,C.S. (2010) Reconstitution of the *B. subtilis* replisome with 13 proteins including two distinct replicases. *Mol. Cell*, **37**, 273-281.
38. Glover,B.P. and McHenry,C.S. (2001) The DNA polymerase III holoenzyme: An asymmetric dimeric replicative complex with leading and lagging strand polymerases. *Cell*, **105**, 925-934.
46. Cull,M.G. and McHenry,C.S. (1995) Purification of *Escherichia coli* DNA polymerase III holoenzyme. *Methods Enzymol.*, **262**, 22-35.
47. Bradford,M.M. (1976) A rapid and sensitive method for the quantitation of microgram quantities of protein utilizing the principle of protein-dye binding. *Anal Biochem*, **72**, 248-254.
